# Likely Native Cases of Leprosy Due to *Mycobacterium lepromatosis* in the United States and Canada

**DOI:** 10.4269/ajtmh.26-0311

**Published:** 2026-06-02

**Authors:** Xiang Y. Han

**Affiliations:** Department of Laboratory Medicine The University of Texas MD Anderson Cancer Center Houston Texas USA E-mail: xhan@mdanderson.org

Dear Editor,

I read with interest the report by Olson et al.^1^ of a patient with leprosy due to *Mycobacterium lepromatosis* in the Pacific northwestern United States. The lack of travel history in this 75-year-old patient and diagnosis in 2021 raise the likelihood of recent autochthonous transmission of the leprosy agent in the northern United States and/or Canada. A similar case diagnosed in 2004 in a 74-year-old man, born and raised in central Canada and without travel to endemic areas, also indicated this possibility.^2^ Recently, paleogenomic studies of millennia-old human skeletons from the Pacific coast of Canada, Chile, and Argentina showed detection of *M. lepromatosis* to suggest the long presence of leprosy in the Americas.^3^^,^^4^

Here, I present two additional cases of likely indigenous infection due to *M. lepromatosis* in the southern United States and Canada. The first is a historic case that occurred in central Louisiana in the 1950s.^5^ The patient was a 52-year-old African American man who had never been out of Louisiana. The eventual diagnosis of diffuse lepromatous leprosy with Lucio phenomenon was based on typical clinical and pathologic features. We now understand that this type of leprosy, also simply called Lucio leprosy, is caused by *M. lepromatosis*.^6^^,^^7^ Louisiana used to be a stronghold for leprosy a century ago. However, this state is far from Mexico, particularly central and Pacific costal Mexico, where Lucio leprosy has been endemic since initial description in 1852 by Lucio and Alvarado.^8^^,^^9^

The second case was presented via email exchange (personal communication, Public Health Agency of Canada, May 2008), about 6 months after our deposition of several *M. lepromatosis* genes to GenBank.^6^^,^^10^ The patient was a Canadian man who presented with dementia and progressed to death rapidly. Numerous acid-fast bacilli were seen in the cerebrospinal fluid, but cultures were negative. The *hsp-65* gene of the organism was amplified for several hundred nucleotides, which yielded a confident identification of *M. lepromatosis* with a 100% match. However, due to the lack of permission from the laboratory leadership, further information on the case, such as the patient’s age, disease course, and travel history could not be obtained. Now, in view of the clinical significance of this leprosy agent and revelation of its long presence in the Americas, I encourage the primary team to fully present the Canadian case after 18 years.
